# Supplementary material for: Social influences on physical activity for establishing criteria leading to exercise persistence
Source: PLoS One. 2022 Oct 19;17(10):e0274259. doi: 10.1371/journal.pone.0274259 (PMC9581432; doi:10.1371/journal.pone.0274259)
Supplement: S1 Appendix — (DOCX) [file pone.0274259.s001.docx]

**APPENDIX**

**DETAILED MODEL DESCRIPTION**

1. **MODEL DEVELOPMENT**

In this model we used a susceptible-infective-susceptible or SIS model framework ^1^ to develop a system of differential equations that describe interactions and transitions between “extremely active”, “moderately active” and “sedentary” population groups. The “extremely active” population group consists of individuals who exercise at least 60 minutes per day, the “moderately active” population group consists of individuals who exercise between 30 and 60 minutes per day and the “sedentary” population group consist of individuals who exercise less than 30 minutes per day. A flow chart describing movement between these compartments appear in manuscript as **Figure 1**. Each compartment represents a population group and **Table 1** in the manuscript lists each term of the differential equation that describes mobility from and to each compartment as shown in manuscript **Figure 1**.

*Model Assumptions*

Every mathematical model is developed using assumptions. Some assumptions are stated to enhance model tractability and are referred to as *simplifying assumptions.* The remaining assumptions are first-principle and are referred to as *mechanistic assumptions*. The following list outlines all the simplifying and mechanistic assumptions that underly our model.

(A1) A constant population can be compartmentalized as follows where the active individuals or exercisers are considered “infected”

- $S\left( t \right)$ Individuals with few exercise habits at week $t$ (susceptible).
- $E_{1}\left( t \right)$ Individuals who are moderately active at week $t$(infected).
- $E_{2}\left( t \right)$ Individuals who are extremely active at week $t$ (infected).

(A2) Social interactions between compartments are governed by the law of mass action and are modeled as a product of population numbers in each compartment.

(A3) Mobility between compartments occurs both from (i) social interaction and (ii) non-social factors.

(A4) Social interactions between members of the exposed compartment (i.e., mobility between $E_{1}$ to $E_{2}$ and vice versa) is modeled linearly.

(A5) A fraction of the population will transition from susceptible to exposed due to non-social factors. This transition is modeled linearly.

(A6) Exposed individuals may transition to the susceptible compartment as a result of social interactions or due to non-social factors (e.g. due to an injury). Social interactions are modeled through the law of mass action while non-social transition is modeled linearly.

We formulate the assumptions (A1)-(A6) using a system of three differential equations and arrive at the following system of equations:

$$S^{'}\left( t \right)=\beta_{1}\frac{E_{1}\left( t \right)S\left( t \right)}{N}-r_{1}\frac{S\left( t \right)E_{1}\left( t \right)}{N}-r_{2}\frac{S\left( t \right)E_{2}\left( t \right)}{N}-\gamma_{1}S\left( t \right)+\beta_{2}\frac{S\left( t \right)E_{2}\left( t \right)}{N}-k_{1}\frac{S\left( t \right)E_{1}\left( t \right)}{N\left( t \right)}-k_{2}\frac{S\left( t \right)E_{2}\left( t \right)}{N}-\gamma_{2}S\left( t \right)+\delta_{1} E_{1}\left( t \right)+\delta_{2}E_{2}\left( t \right)$$

$${E_{1}}^{'}\left( t \right)=r_{1}\frac{S\left( t \right)E_{1}\left( t \right)}{N}+r_{2}\frac{S\left( t \right)E_{2}\left( t \right)}{N}+\gamma_{1}S\left( t \right)-\beta_{1}\frac{E_{1}\left( t \right)S\left( t \right)}{N}-\alpha_{2}E_{1}\left( t \right)+\alpha_{1}E_{2}\left( t \right)-\delta_{1}E_{1}\left( t \right)$$

$${E_{2}}^{'}\left( t \right)=k_{1}\frac{S{\left( t \right)E}_{1}\left( t \right)}{N}+k_{2}\frac{S{\left( t \right)E}_{2}\left( t \right)}{N}+\gamma_{2}S\left( t \right)-\alpha_{1}E_{2}\left( t \right)+\alpha_{2}E_{1}\left( t \right)-\beta_{2}\frac{E_{2}\left( t \right)S\left( t \right)}{N}-\delta_{2}E_{2}\left( t \right)$$

Subject to three non-negative initial conditions: $S\left( t=0 \right)= S_{0}$, $E_{1}\left( t=0 \right)= E_{1}^{0}$ and $E_{2}\left( t=0 \right)= E_{2}^{0}$.

(A7) We are interested in investigating the time-dependent changes in active and sedentary groups within a fixed population, hence we impose the restriction that the total number of individuals $N\left( t \right)$ remains constant and is defined as the sum of the susceptible and exposed individuals $N\left( t \right)=N= S\left( t \right)+E_{1}\left( t \right)+ E_{2}\left( t \right).$

Assumption (A7) is consistent with the above system of differential equations provided that $\frac{d}{dt}\left( S\left( t \right)+E_{1}\left( t \right)+ E_{2}\left( t \right) \right)=0,$which holds when the total number of individuals entering West Point equals the total number of individuals leaving West Point. We refer to the above system of equations as **System 1.** A full description of each term in **System 1** appears in **Table 1**. A flow chart of the movement between compartments appears in **Figure 1**.

1. **DESCRIPTION OF THEORETICAL RESULTS**

The solutions to **System 1** exist, are non-negative, and bounded. This is easily proved and we refer the reader to the article by Thomas et al. in ^2^ for an analogous rigorous proof to this statement.

Because we have objective information on physical activity data at the United States Military Academy (USMA), we will use USMA data to estimate parameters and thresholds required to draw individuals to an active lifestyle. For our simulations and to derive meaningful parameter estimates, we will assume that $\gamma_{1,2}=0$. This means that we are assuming all physical activity changes in the sedentary population are due to social interaction. After making this assumption, we can determine the range of parameter values $r_{1,2},k_{1,2},\beta_{1,2},\alpha_{1,2},\delta_{1,2},S_{0},E_{1}^{0},E_{2}^{0}$that will draw more individuals to increase their weekly physical activity. This range is known as a Threshold Theorem and depends on the value of the parameter $R_{0}$ stated “R naught”. This value is known as the basic reproduction number, which represents the average number of secondary infections produced by a typical infection in a population where everyone is susceptible. If $R_{0}<1,$ the infection dies out; whereas if $R_{0}>1$, the viral infection spreads through the population. We are interested in the combination and range of model parameters that yield a basic reproduction number $R_{0}>1.$

**Proposition 2**: *Threshold Theorem*

*Given parameters* $k_{1,2}, r_{1,2}{, \alpha}_{1,2},\beta_{1,2}, {\delta_{1,2},S}_{0},E_{1}^{0},E_{2}^{0}$*, healthy lifestyle by means of exercise will spread throughout the population if the basic reproduction number,*$R_{0},$ *has the property that* $R_{0}>1.$*If* $R_{0}<1$ *then the healthy lifestyle habits amongst the population will “die out”. The value of* $R_{0}$ *is given by:*$R_{0} = \frac{B + \sqrt{B^{2}-4AC}}{2A}$*, where A B and C are combinations of the parameters* $k_{1,2},r_{1,2}{, \alpha}_{1,2},\beta_{1,2},\delta_{1,2},S_{0},N$ *as follows:*

$$A=N^{2}\left( \alpha_{1}\delta_{1}+\delta_{2}\left( \alpha_{2}+\delta_{1} \right) \right)+NS_{0}\left( \beta_{1}\left( \alpha_{1}+\delta_{2} \right)+\beta_{2}\left( \alpha_{2}+\delta_{1} \right) \right)+{S_{0}^{2}\beta}_{1}\beta_{2}$$

$$B={S_{0}[N (\alpha}_{1}k_{1}+\delta_{1}k_{2})+N (\alpha_{2}\left( k_{2}+r_{2} \right)+r_{1}\left( \alpha_{1}+\delta_{2} \right))+S_{0} \left( \beta_{1}k_{2}+\beta_{2}r_{1} \right)]$$

$$C=\left( r_{1}k_{2}-r_{2}k_{1} \right)S_{0}^{2}$$

We use the next generation matrix method as outlined in ^3^ and split the terms in the exposed compartments into two categories:

1. The rate of appearance of new active individuals (new infections) in compartment *i*, denoted by $\mathcal{F}_{\mathcal{i}}.$
2. The rate of transfer of individuals into compartment $i$ by all other means (other than newly infected), denoted by $\mathcal{V}_{\mathcal{i}}^{+}$. The rate of transfer of individuals out of compartment $i$, denoted by $\mathcal{V}_{\mathcal{i}}^{-}$ .
3. The terms defined in (ii) can be combined to $\mathcal{V}_{\mathcal{i}}=\mathcal{V}_{\mathcal{i}}^{-}-\mathcal{V}_{\mathcal{i}}^{+}$

$R_{0}$ is the largest eigenvalue of the matrix obtained by forming the product of the Jacobian matrix $F =\frac{\partial\mathcal{F}_{i}}{\partial x_{j}}$ and the inverse of the Jacobian matrix $V =\frac{\partial\mathcal{V}_{i}}{\partial x_{j}}$ (i.e., $R_{0} = \rho\left( FV^{-1} \right)$), both evaluated at the disease free equilibrium (DFE). The disease free equilibrium takes the form of $\left( S_{0,}0,0 \right)^{T}.$ Evaluating F and V at the disease free equilibrium point, we obtain:

$F = \frac{S_{0}}{N}\left( \begin{matrix} r_{1} & r_{2} \\ k_{1} & k_{2} \end{matrix} \right)$ and $V = \left( \begin{matrix} \alpha_{2}+\beta_{1}\frac{S_{0}}{N}+\delta_{1} & -\alpha_{1} \\ -\alpha_{2} & \delta_{2}+\alpha_{1}+\beta_{2}\frac{S_{0}}{N} \end{matrix} \right)$.

The largest eigenvalue leads to the threshold parameter, which takes the form:

$$R_{0} = \frac{B + \sqrt{B^{2}-4AC}}{2A},$$

where A B and C are combinations of the parameters $k_{1,2},r_{1,2}{, \alpha}_{1,2},\beta_{1,2},\delta_{1,2},S_{0},N$ as follows:

$$A=N^{2}\left( \alpha_{1}\delta_{1}+\delta_{2}\left( \alpha_{2}+\delta_{1} \right) \right)+NS_{0}\left( \beta_{1}\left( \alpha_{1}+\delta_{2} \right)+\beta_{2}\left( \alpha_{2}+\delta_{1} \right) \right)+{S_{0}^{2}\beta}_{1}\beta_{2}$$

$$B={S_{0}[N (\alpha}_{1}k_{1}+\delta_{1}k_{2})+N (\alpha_{2}\left( k_{2}+r_{2} \right)+r_{1}\left( \alpha_{1}+\delta_{2} \right))+S_{0} \left( \beta_{1}k_{2}+\beta_{2}r_{1} \right)]$$

$$C=\left( r_{1}k_{2}-r_{2}k_{1} \right)S_{0}^{2}$$

The above simulation can be simplified for cases in which interactions between compartments are limited. Specifically, we look at the following cases:

1. There are no social interactions between sedentary and moderately individuals and the spontaneous recidivism rate of all compartments are zero. This can be achieved by setting the social transition rates $r_{1}=k_{1}=\beta_{1}=0,$ and spontaneous movement parameters $\gamma_{1} = \gamma_{2} = 0 \& \delta_{1} = \delta_{2} = 0.$

$$R_{0}=\frac{k_{2}+r_{2}}{\beta_{2}}.$$

**Figure 2A** shows that the sedentary population becomes extinct over time with the population transitioning to a plateau of exercisers. **Figure 2B** illustrates that if the sum of the social parameters $k_{2}$ and $r_{2}$ are lower than $\beta_{2}$ the exercising populations become extinct and sedentary behavior persists.

1. There are no social interactions between sedentary and extremely active individuals and there is no spontaneous movement from extremely active to sedentary compartments. Transition rates: $r_{2}=k_{2}=\beta_{2}=0 \& \delta_{2}= 0$

$$R_{0}=\frac{{(k}_{1}+r_{1})\frac{S_{0}}{N}}{\beta_{1}\frac{S_{0}}{N}+\delta_{1}}.$$

**Figure 3A** shows that the sedentary population becomes extinct over time with the population transitioning to a plateau of exercisers. **Figure 3B** illustrates the case where exercising populations become extinct and sedentary behavior persists.

1. Suppose that there are no social interactions between the sedentary and moderately active groups. Additionally, extremely active individuals do not transition to the sedentary compartment (no injuries among extremely active). Mathematically, we set $r_{1}=k_{1}=\beta_{1}={0, \gamma_{1} = \gamma_{2} = 0, \delta}_{2}=0$. The parameter $R_{0}$ takes the following form:

$$R_{0}=\frac{{\frac{S_{0}}{N}(\alpha_{2}(k}_{2}+r_{2})+k_{2}\delta_{1})}{{\frac{S_{0}}{N}(\beta_{2}(\alpha}_{2}+\delta_{1}))+\alpha_{1}\delta_{1}}.$$

In this case, we see that the threshold parameter depends on the social interactions that draw sedentary individuals to the exercise compartments (such as $k_{2},r_{2}$), the social interactions that drive movement between the active compartments ($\alpha_{1}$ and $\alpha_{2}$) and the recidivism parameters that draw the exercisers to a sedentary lifestyle. **Figure 4A** shows the simulations that lead to the prevalence of exercise and **Figure 4B** shows the simulations that lead to the prevalence of sedentary habits.

**Proposition 3:** *The mathematical model (System 1) yields to a steady state. If the physical activity prevalence arrives at this value, it remains at this value.*

We refer the reader to the article by Thomas et. al. in^2^, for a theoretical proof of Proposition 3.

**Appendix Figure 1.** Dependency of the sedentary population on the social interaction parameters $k_{1},r_{1},\gamma_{1}{,\delta}_{1},\beta_{1}$. **Panel A** illustrates the evolution of the sedentary population over 1500 weeks plotted for ten different transition rates ranging from $k_{1}=0.001 to k_{1}=0.01$ where $k_{1}$ represents the fraction of sedentary individuals who become extremely active as a result of interactions with moderately active individuals. **Panel B** shows the percent of sedentary population for ten transition rates ranging from $r_{1}=0.001 to r_{1}=0.01$ where $r_{1}$ represents the fraction of sedentary individuals who become moderately active as a result of interacting with moderately active individuals. **Panel C** illustrates the percentage of sedentary population over time as the transition parameter $\gamma_{1}$ varies from $\gamma_{1}=0.001 to\gamma_{1}=0.01$ where $\gamma_{1}$ represents the fraction of sedentary individuals who spontaneously transition to moderately active. **Panel D** illustrates the evolution of percentage of sedentary population over time as the transition parameter $\delta_{1}$ varies from $\delta_{1}=0.001 to\delta_{1}=0.01$ where $\delta_{1}$ represents the fraction of moderately active individuals who spontaneously transition to sedentary (e.g., as result of injury). Panel E illustrates the percentage of sedentary population over time as the social transition parameter $\beta_{1}$ varies from $\beta_{1}=0.001 to\beta_{1}=0.01$ where $\beta_{1}$ represents the fraction of moderately active individuals who transition to sedentary as a result of social interactions with sedentary individuals.

| 1. Increasing social interactions between sedentary and extremely active individuals (increase social interaction rate $k_{2}$ that moves sedentary to extremely active) decreases the long-term plateau of sedentary population by 16%.   **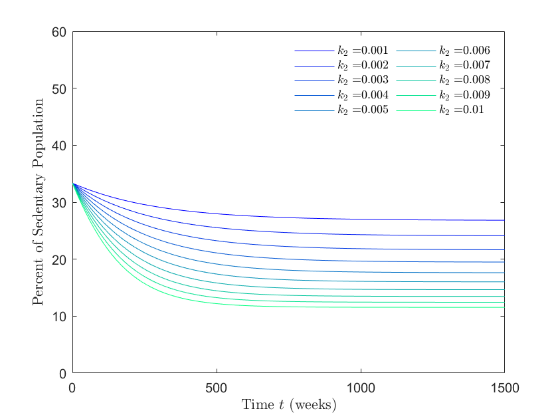** | 1. Increasing social interactions between sedentary and extremely individuals (increase social interaction rate $r_{2}$ that moves sedentary to moderately active) decreases the long-term plateau of the sedentary population by 13%.   **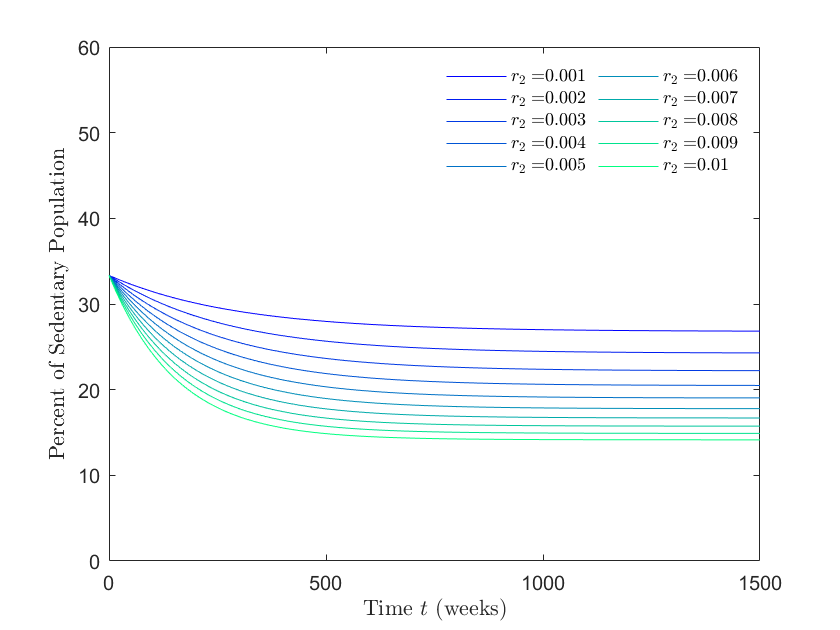** |
| --- | --- |
| 1. Increasing spontaneous movement parameter from sedentary to extremely active ($\gamma_{2}$) decreases the long-term plateau of sedentary population by 20 %.   **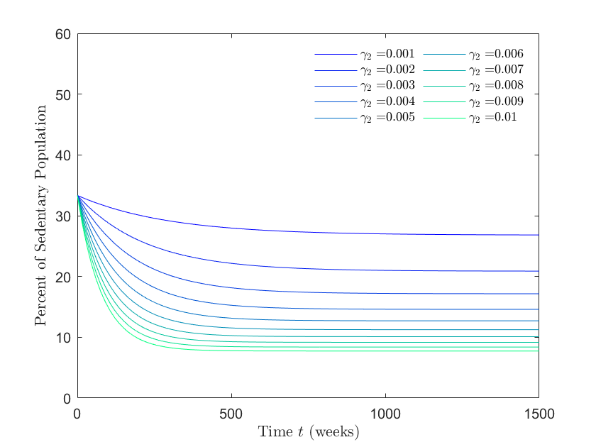** | 1. Increasing spontaneous movement parameter ($\delta_{2}$) from extremely active to sedentary decreases the long-term plateau of sedentary population by 23 %.   **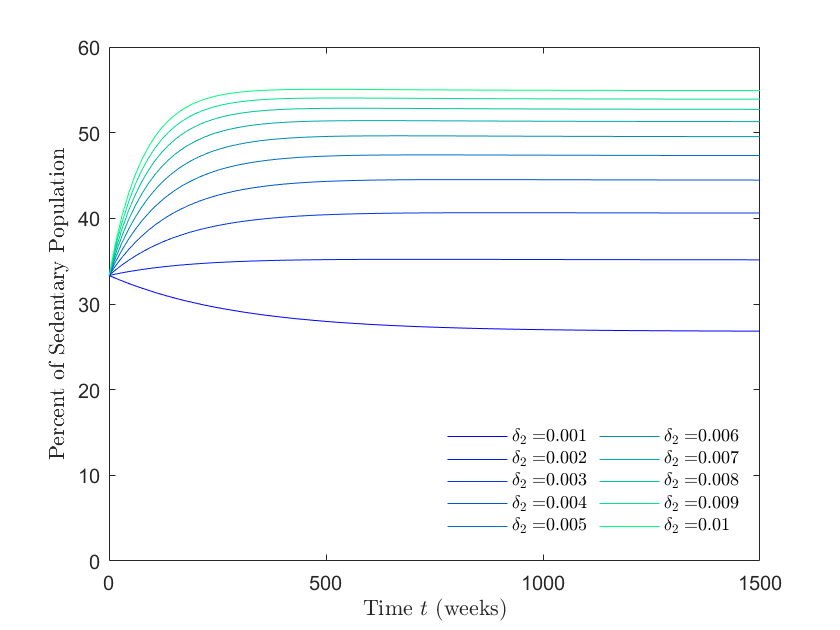** |
| 1. Increasing social interactions between sedentary and extremely active individuals (increase recidivism rate $\beta_{2}$) increases the long-term plateau of the sedentary population by 21%.   **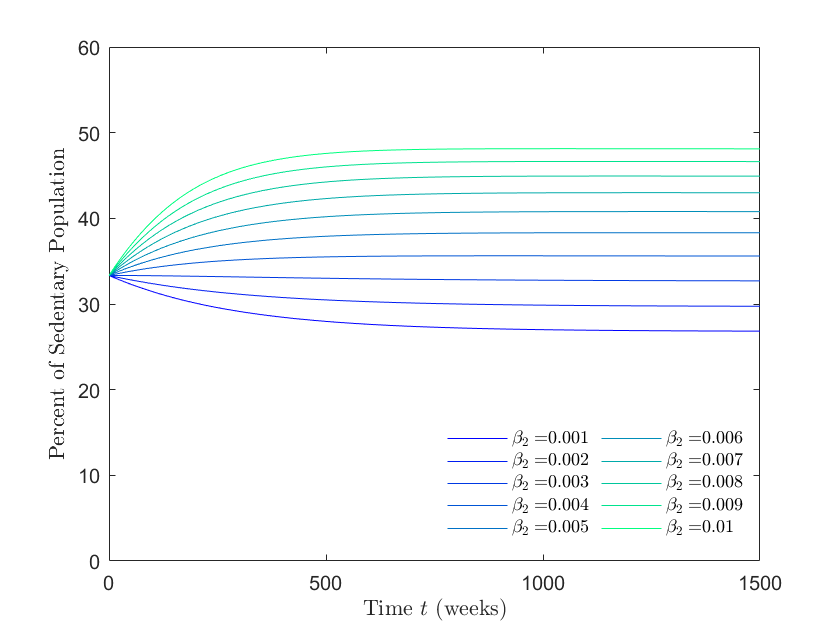** | |

**Appendix Table 1** Summary characteristics of Army Physical Fitness Test Run Time by USMA Academic Department

| **Academic Unit (N)** | **Age (years)** | **Run Time (seconds)** |
| --- | --- | --- |
| AB (32) | 36.97±5.53 | 886.66±200.28 |
| AF (25) | 36.92±5.69 | 789.36±306.17 |
| AH (36) | 36.53±4.91 | 841.33±97.09 |
| AJ (22) | 36.68±5.52 | 849.36±109.42 |
| F (21) | 40.00±5.97 | 993.19±96.63 |
| I (23) | 39.17±5.92 | 959.65±339.40 |
| N (20) | 41.60±5.16 | 976.10±253.56 |
| P (22) | 38.50±7.17 | 899.68±101.49 |
| Q (30) | 39.83±5.55 | 1007.20±406.28 |
| S (27) | 38.67±5.92 | 938.67±86.16 |
| T (24) | 39.25±6.39 | 936.46±208.49 |
| V (9) | 43.11±4.96 | 1007.33±138.68 |
| W (42) | 35.86±4.38 | 908.62±277.74 |
| Y (17) | 35.88±5.89 | 769.41±208.41 |
| Z (22) | 37.59±6.12 | 909.18±115.16 |

**References**

1. Kermack WO, McKendrick AG. A contribution to the mathematical theory of epidemics. *Proc R Soc Lond A* 1927;115:700–721.

2. Thomas DM WM, Fuemmeler BF, et al. Dynamic model predicting overweight, obesity and extreme obesity prevalence trends. *Obesity (Silver Spring).* 2014:590-597.

3. Driessche Pvd. Reproduction numbers of infectious disease models. *Infectious disease modeling.* 2017:288-303.
