# Supplementary material for: Social influences on physical activity for establishing criteria leading to exercise persistence
Source: PLoS One. 2022 Oct 19;17(10):e0274259. doi: 10.1371/journal.pone.0274259 (PMC9581432; doi:10.1371/journal.pone.0274259)
Supplement: Attachment — Submitted filename: Response to Reviewer 2.docx [file pone.0274259.s003.docx]

**Response to Reviewer 1**

We thank the reviewer for spending the time to carefully go through the manuscript again. We have responded to each comment and any change made in the manuscript has been copied and pasted in red for the convenience of the reviewer.

Reviewer #1: The report presents the outcome of a study on the social influence on physical activity in a community. The contribution of the report to the body of knowledge is significant and novel. Also, the aim and objectives of the study are within the scope of your journal. However, the present form of the report needs revision. The author should consider the following points:

**Comment Number 1:** The title presents something impossible. How is it possible that dynamic model of social has influence on physical activity?

Revise the title or consider:
Social influences on physical activity for establishing criteria leading to exercise persistence

**Response to Comment Number 1:** We were trying to get in that this was a mathematical model, but agree with the reviewer that it turns out to a mouthful of nothing. We liked the title the reviewer suggested and changed our title to the suggested title.

**Comment Number 2:** Line 42, it was written:
Objectives: The objective of this study is to dynamically model social influences on physical activity to establish quantitative criteria that lead to sustained exercise habits.
Comment: Revise the objectives to present the objectives of the research for social influences on physical activity for establishing criteria leading to exercise persistence

**Response to Comment Number 2:** We have changed the objective as suggested:

The objective of this study is for establishing criteria for social influences on physical activity for establishing criteria that lead to exercise persistence.

Comment Number 3 & 4: It is now necessary to delete the headings under the abstract like: Background:, Objectives:, Methods:, Results:, and Conclusions:. Ensure that the abstract is not up to 250 words.

Response to Comment Number 3 & 4: We have removed the structured portion of the abstract. The word count is 229 words.


**Comment Number 5:** The authors did not respond to. Update the report with a published fact on mathematical modeling. The author may consider, “According to Ref A et al. (2022), mathematical modeling involves analyzing real-world situations using mathematical terminology and involves transforming them into a mathematical form.”
Ref A et al. (2022): Ratio of Momentum Diffusivity to Thermal Diffusivity: Introduction, Meta-analysis, and Scrutinization. Chapman and Hall/CRC. New York, 2022. ISBN-13: 978-1032108520, ISBN-10: 1032108525, ISBN9781003217374

Response to Comment Number 5: We did in fact respond to this. We referenced a seminal mathematical modeling book that we use to teach at West Point. The textbook includes the mathematical modeling triangle that starts from transform form a real world problem to solve the mathematical formulation to interpret the solution. However, we have now included the suggested reference as well. The paragraph is below with the suggested reference as Number 24.

Mathematical model development requires transformation of insights and data from to a mathematical formulation followed by solving or simulating the mathematical formulation. After solving/simulating interpretation of the simulations are needed to nest the findings back to the real-world scenario[23, 24]. The description of our model development follows the “math modeling triangle” process described in [23].

**Comment Number 6:** The revised version is difficult to understand. Remove the first version. Delete the old sentence. You just need to highlight the revised path. Do not underline any sentence.

**Response to Comment Number 6:** We understand, however, the PLoS One guidelines indicated we needed to use track changes which is what we have done. We have the clean version and the snippets cut here so that the reviewer can read them. We have also highlighted changes and supplied the highlighted version as supplemental materials for the reviewer only.

**Comment Number 7:** Line 417, it was written:
We hope that our model and results will inspire future experimental investigations in community intervention research that may provide further insight and lead to improved recommendations.

One of the most crucial parts of the manuscript is the conclusion. By examining the key ideas in the report, bringing these ideas to a convincing conclusion, and presenting a concluding viewpoint, it makes a lasting effect on the reader. The authors should try to give the reader a feeling of closure with a concluding sentence. This is needed to boost the impact of authors claim. A sentence is needed to first re-present the aim of the study elaborately. Then remark that the major objectives had been established. Start the conclusion section with a fact on the achievement of the research aim before stating conclusive statements. Revise the title to present the aim concisely. The aim of the title should reflect in the abstract, and at the beginning of the conclusion. Author's should revise the conclusion section to provide conclusive statements on the research questions posed at the end of the introduction. The conclusion section should be revised.

**Response to Comment 7:** We have revised to conclusion to match the title and explain what we found in context of the community interventions:

Here. we have established criteria on social influences that lead to exercise persistence. Specifically, we found that the social interaction between moderately active individuals with the sedentary population should be engineered to draw the sedentary individuals into more active behaviors. A second criteria identified that strategies are needed to control dropouts from moderately active individuals. Finally, we found that discouraging socially acceptable sedentary behavior will reduce drop out of moderately active individuals. These findings can be used to design and implement community based exercise programs that lead to long-term exercise persistence in the community.
